# Use of Simultaneous Bilateral Cataract Surgery (SBCS) to Optimize Parameters Affecting the Subjective Perception of the Procedure

**DOI:** 10.1155/2021/5584906

**Published:** 2021-06-11

**Authors:** P. Studený, J. Vránová, L. Nováček

**Affiliations:** ^1^Department of Ophthalmology, University Hospital Kralovske Vinohrady and 3rd Faculty of Medicine, Charles University, Prague, Czech Republic; ^2^Eye Clinic Somich, Karlovy Vary, Czech Republic; ^3^Department of Medical Biophysics and Informatics, 3rd Faculty of Medicine, Charles University, Prague, Czech Republic; ^4^Department of Ophthalmology, The Institute of Aviation Medicine, Prague, Czech Republic

## Abstract

**Introduction:**

Simultaneous Bilateral Cataract Surgery (SBCS) is still a relatively controversial procedure. The main objection is the risk of bilateral endophthalmitis or bilateral refractive error. However, SBCS has also some advantages (faster visual rehabilitation, lower risk of nosocomial infection, and lower cost). Performing surgery on both eyes in one session has one additional advantage which has not yet been described in the literature (according to the information available to authors). It allows surgeons to distinguish the effect of minor differences in the surgical protocol on the subjective perception of the procedure more accurately, which is a more suitable method than comparing two independent groups of patients.

**Purpose:**

To compare the effect of minor changes in the surgical protocol during SBCS on intraindividual subjective perception of surgery (pain, pressure, glare, and perception of the duration of the surgery).

**Methods:**

During the surgery of the right and left eyes of one patient, we randomly changed one surgical parameter (use of intracameral anesthesia, light intensity of the operating microscope, type of eyelid speculum, creation of the posterior circular capsulorhexis, and communication with the patient during surgery). Patients immediately after both surgeries subjectively evaluated the perception of pain (on the scale 0–10), pressure, and glare (on the scale 0–5) and estimated the duration of the surgery, separately for each eye. Each change was evaluated in a group of 50 patients.

**Results:**

In the control group with no parameters changed, we noted no difference in subjective perception of the first and second surgery. In subgroups, where we changed the protocol, we detected only minor differences in subjective perception of pain, pressure, glare, and duration of the surgery. Only one statistically significant difference in subjective pain perception was in the subgroup where we used eye intracameral anesthesia (0.34 eyes with intracameral anesthesia, 0.44 eyes with only topical anesthesia). We did not note any statistically significant differences in the perception of the time of surgery.

**Conclusion:**

SBCS can be used to optimize the parameters of cataract surgery. In our study, we noted a positive effect of intracameral anesthesia on subjective perception of surgery.

## 1. Introduction

Simultaneous Bilateral Cataract Surgery (SBCS), in the literature also known as Immediately Sequential Bilateral Cataract Surgery (ISBCS), is still a relatively controversial practice in the treatment of bilateral cataract surgery. The main opponents' argument is the risk of bilateral blindness due to severe postoperative complications, such as endophthalmitis, toxic anterior segment syndrome, or pseudophakic bullous keratopathy [1–5]. However, it has been repeatedly reported that, with careful selection of patients and strict adherence to the protocol, this risk is minimal [6, 7].

Another argument against this is the impossibility of adjusting the calculation of the value of the intraocular lens (IOL) in the case of refractive deviation in the first operated eye [8].

On the other hand, the SBCS has a number of advantages over the Delayed Sequential Bilateral Cataract Surgery (DSBCS), for instance, the decrease in waiting time for surgery, faster rehabilitation, patient convenience, and time savings and cost savings for healthcare providers [9–12].

However, SBCS brings another little-mentioned advantage for cataract surgery research. Performing surgery on both eyes during one stay in the operating room allows the patient to compare subjectively the course of surgery on each eye. In the case of a minor change in the surgical protocol during surgery on one and the other eye of the same person (within the limits of the guidelines), the patient can evaluate the effect of such change on subjective perception. In the case of delayed surgery of both eyes, it is very difficult for the patient to notice small changes in subjective perception. Researchers had to create two large groups of patients to compare the influence of change. In the case of the method described by us, the patients subjectively compare the difference in one and the other eye (intraindividually). This minimizes selection bias.

This study aims to compare the effect of minor changes in the surgical protocol (use of intracameral anesthesia, light intensity of the operating microscope, type of eyelid holder, creation of the posterior circular capsulorhexis, and communication with the patient during surgery) on intraindividual subjective perception of surgery (pain, pressure, glare, and perception of the duration).

## 2. Participants and Methods

The present study was conducted as a prospective, monocentric, randomised, blinded study. It was performed at the Somich eye clinic, Karlovy Vary, Czech Republic. Patients with bilateral cataracts who had uneventful SBCS in the period between July 2019 and March 2020 at the Somich eye clinic were consecutively enrolled. The exclusion criteria were amblyopia of one eye, the difference of the planned value of the implanted intraocular lens between both eyes greater than 1.5 D, previous intraocular surgery, a history of eye injury, severe fundus pathology, severe corneal scars, and perioperative complications. The study protocol was approved by the Ethics Committee following the Declaration of Helsinki. All participants signed an informed consent form before participation in this study.

### 2.1. Surgical Technique

All surgeries were done by one high volume surgeon (PS), and both surgeries were performed as two separate interventions during one patient stay in the operating room. The protocol of surgery meets the recommendation of the International Society of Bilateral Cataract Surgeons [13]. We always performed right eye surgery first, and then left eye. Immediately after the surgery, patients received standard eyedrop medication with antibiotic and nonsteroidal anti-inflammatory drops, and eyes were left uncovered. The nurse recorded the exact time of both surgeries from the time the speculum was put on and after it was removed again.

### 2.2. Assessment

After undergoing surgery on both eyes and leaving the operating room, patients completed a simple questionnaire with the help of a nurse who read the questions to the patients and wrote down their answers. In our questionnaire on the numerical scale, the patients expressed pain (0–10, 0 no pain, 10 maximum unsustainable pain), perception of pressure (0–5, 0 no pressure, 5 maximum pressure), and feeling of glare (0–5, no problem with glare, 5 maximum glare) and they also estimated the duration of the surgery.

### 2.3. Groups of Patients

At first, we examined 50 patients (100 eyes) with no change between the two surgeries, to see if subjects perceived a subjective difference in both eye surgeries, for instance, depending on whether it was the first or second eye surgery (control subgroup).

Then, we gradually changed these parameters of the surgery; for each changed parameter, we created a group of 50 patients (100 eyes):

Anesthesia—in one eye we used only topical anesthesia, in the other eye additionally was administered 0.02 ml of Mydrane (Phenylephrine hydrochloridum, Tropicamidum, Lidocaini hydrochloridum), (Laboratoires Thea, Clermon Ferrand Cedex 2, France)—subgroup 1.

Light intensity of the surgical microscope—OPMI Lumera 700 (Carl Zeiss Meditec AG, Jena, Germany), in one eye illumination was 40% of the maximal intensity, in the other eye 80%—subgroup 2.

Speculum (Lieberman type—both) with a solid (closed) blade in one eye and wire (open) blade speculum in the other—subgroup 3.

Creation of the primary posterior circular curvilinear capsulorhexis (PPCCC)—in one eye was left posterior capsule intact, in the other eye we made PPCCC—subgroup 4.

Communication during surgery—during the operation of one eye, the surgeon communicated with the patient practically all time, and during the procedure of the other eye, the communication between the surgeon and patient was minimal—subgroup 5.

The changed parameter was used randomly on the right or left eye. The patient was not informed either of which parameters were changed or in which eye.

### 2.4. Statistical Analysis

Statistical analysis was performed using SPSS Statistics software (version 21, IBM Corp.) The comparison was performed by Student's paired *t*-test in the case of normal distributed variables and nonparametric Wilcoxon's test in the case of nonnormal distributed variables. A *p* value less than 0.05 was considered statistically significant.

## 3. Results

Table 1 shows the baseline and demographic data. The mean age was 72.5 ± 8.4 years (range 46–93 years). The similar mean age was also in the different subgroups. The small differences were not statistically significant. Number of men in all groups was 114 (38%), and the number of women was 186 (62%). This ratio was similar in all subgroups, apart from subgroup 5 which had the same number of men and women. The mean power of the implanted intraocular lens (IOL) was 21.3 ± 3.2 D and this value was similar in all different subgroups (statistically not significant differences).

In the control group, in which we did not change any parameters during the surgery of the right and left eyes, we did not find any statistically significant difference between the perception of right and left eye surgery. Differences between pain, pressure, and glare perceptions were minimal, statistically not significant (Table 2). We also did not register any difference in the time perception of the surgery duration. The mean real time of surgery was 262 ± 62 seconds on the right eye and 261 ± 71 seconds. The subjectively perceived time of surgery was 259 ± 121 on the right eye and 262 ± 129 on the left eye (Table 3). This means that the patients perceived the duration of surgery on average 8 seconds longer in the right eye and 2 seconds shorter in the left eye, respectively. The difference was not statistically significant.

In the individual subgroups, we did not notice (with one exception) any difference in the perception of pain, pressure, and illumination during surgery between the two eyes. Additionally, the perception of the duration of the surgery did not differ, or the differences were not statistically significant. The only difference in the perception of pain during the surgery was noted in subgroup 1 with a changed type of anesthesia. In the eye where Mydrane was used intracamerally, the mean value of subjectively perceived pain was 0.32 ± 0.71, while in the eye where only superficial anesthesia was used, the value was 0.44 ± 0.81. This difference was statistically significant (Tables 4 and 5).

## 4. Discussion

One of the earliest scientific reports of simultaneous binocular cataract in one operating session was published in 1952 [14], initially applied to ICCE operations. Over the years, surgical techniques were refined and supporters of SBCS appeared. SBCS began to become more common with the rise of small incision phacoemulsification [10]. SBCS is fully accepted in paediatric patients, or otherwise handicapped, especially if the surgeries are performed under general anesthesia. The risk of death during the second general anesthesia statistically exceeds the risk of bilateral blindness due to a serious complication [6], though in general the popularity of SBCS increases in the case of uncomplicated bilateral surgery for age-related cataracts, under topical anesthesia.

At our clinic, we standardly perform 80–90% of procedures with the technique SBCS, so far without any serious complications. Interestingly, some patients spontaneously perceived some differences in their right and left eye surgery, despite the fact that the operation was relatively the same. In the first phase of our study, we therefore tried to register differences in the perception of surgery in both eyes.

In clinical practice, it is a common observation amongst ophthalmic surgeons that patients with bilateral cataracts often report more pain and discomfort during the second consecutive eye surgery (DSBCS). Previous studies have investigated this observation and have also reported an increase in pain in the second surgery relative to the first [15–17]. Yu et al. published the results of subjective sensation of the surgery of both eyes in the group of 127 patients with bilateral cataracts, operated as DSBCS (the interval between surgeries was approximately 1 month). Patients experienced less anxiety, a greater number of eye bulges, and greater pain during the second cataract surgery compared with the first surgery. The differences in the scores for light sensitivity between surgeries were not statistically significant [18]. Adatia et al. in their study described that of 125 patients who rated second eye surgery as the generally more unpleasant procedure, 90 (72.0%) were similarly or more relaxed during the second procedure. Second-eye cataract surgery was perceived as being a longer and/or more painful procedure by a significant number of patients (45.4%) [19]. Finally, Shi et al. published a meta-analysis of seven studies by comparing pain scores, assessed shortly after phacoemulsification under local anesthesia. This analysis showed that there were statistical differences in pain values between the first and the second eye (WMD: 0.69; 95% CI: 0.40, 0.98; *p* < 0.00001; with high heterogeneity (*I*^2^ = 80%). The pain value was lower in patients who underwent the first eye surgery [20]. Ursea et al. hypothesized that a subtle increase in pain in the second surgery relative to the first appears to be associated with decreased preoperative anxiety and may be related to the amnestic effect of intravenous sedation [16]. Also, Jiang et al. and Nijkamp et al. in their studies confirmed that greater pain in the second operated eye is related to less anxiety [21, 22]. However, according to the information available to us, so far, no one has found a difference in the perception of surgery on both eyes if they were performed on the same operation day (SBCS).

In our group of 50 patients (control subgroup), the differences between pain, pressure, and glare perceptions were minimal and statistically not significant. We also did not register any difference in the time perception of the duration of the surgery. This means that if a change is made during the surgery of one of the two eyes, that patient could register such a change in the perception of pain, pressure, glare, or in estimating of the time of surgery. We had selected five minor changes that we perform on our patients if necessary during the surgical protocol and that did not affect the outcome of the operation. In the study, we always performed a change in one of the parameters in a targeted manner in a group of patients, randomly to the first or second operated eye.

The effect of intracameral anesthesia on pain perception during surgery has been published quite frequently. Nebbioso et al. summarized that intracameral administration of lidocaine is a simple and secure method able to increase the analgesia during cataract surgery, eliminating the discomfort and increasing the cooperation of the patients during the steps of manipulation [23]. On the contrary, Crandall et al. did not find a statistically significant difference but described that patients in the lidocaine group reported being less bothered by tissue manipulation. The surgeon assessment also showed more patient cooperation in the lidocaine group [24]. Ezra and Allan published a meta-analysis, a total of eight trials comprising 1281 patients. Their data comparison showed a significantly lower intraoperative pain perception in patient groups using supplementary intracameral lidocaine, although the difference was small. No significant difference was demonstrated between the groups receiving topical anesthesia alone and topical combined with intracameral anesthesia in terms of need for supplemental anesthesia, intraoperative adverse events, or corneal toxicity [25].

In our subgroup of patients with changed types of anesthesia, we noticed a statistically significant difference in the perception of pain. The pain of the surgery (or the difference between the right and left eye) was assessed by the patients in general, after surgery. We did not determine the effect of individual steps. The pain was lower in the eyes, where intracameral anesthesia (Mydrane) was used, in addition to topical anesthesia, in comparison to eyes with topical anesthesia alone, although its use did not affect the perception of pressure, glare, or duration of the surgery.

Relatively few papers have been published on the influence of lighting intensity on the subjective perception of surgery. Most patients perceive a light sensation during surgery, while many of them describe this sensation as unpleasant. Biró and Schvöller in their prospective study noted that every patient saw lights of different and changing intensity. One hundred and twenty-six (95.5%) patients saw different colours; 13.6% patients saw a rainbow-like scale of colours; 74.2% patients could see clear shapes and forms (mainly circle, square, rectangle, and ellipse). Forty-nine (37.1%) patients could see the instruments, and 35 (26.5%) patients saw the fingers of the surgeon during surgery. Twenty-six patients (19.7%) considered the strong light of the microscope very disturbing and uncomfortable. The authors found no relation between intraoperative visual sensation and patient's age, sex, preoperative visual acuity, duration of surgery, or cataract severity [26]. Kim et al. compared the microscope illumination and intracameral illumination. They concluded that the microscope ocular illuminance and irradiance during cataract surgery were higher in the microscope illumination than in the intracameral illumination. It suggests that light exposure reaching the patient's and surgeon's retina during cataract surgery is lower in the intracameral illumination than in the microscope illumination [27]. A similar work with the same conclusion was also published by Seo et al. The authors summarized that, in view of the patients' visual experience, oblique intracameral illumination caused less subjective photostress and was preferred over coaxial microscope illumination [28].

In our group of patients, in which we operated with 40% microscope intensity illumination in one eye and in the other eye with 80%, we did not observe any effect on the subjective perception of pain, pressure, or duration of the operation. Surprisingly, the patients did not notice any greater difference in the degree of glare. In the eye with 40%, the mean described glare was 0.82 ± 1.10, in the eye with 80% 0.86 ± 1.11, but the difference was not statistically significant.

The effect of cataract density can rather be ruled out, as only patients with approximately symmetrical cataracts in both eyes were included into the study.

Very few papers have also been published on the effect of speculum on the subjective perception of surgery. Crosby et al. investigated the effect of eyelid holder on postoperative ptosis. They concluded that the different speculae have significantly different forces on patients' eyelids during surgery. The patients who experience the greatest compression of the speculae are those with the smallest palpebral apertures. This may explain why these patients are more likely to develop postoperative lid malposition [29]. The fact that the type of speculum has a different effect on the condition of the eye during surgery is apparent, for example, from the work of Lagetá Queiroz et al. They concluded that the speculum affects the spherical equivalent, measured by aberrometry readings. The open blade speculum was the most similar to the data captured without the speculum [30]. Raevis et al. compared the effect of the speculum on pain perception during intravitreal injection. The authors compared the speculum and cotton-tipped applicator and the unimanual technique of application. They concluded that unimanual and cotton-tipped methods are significantly less painful [31].

While we can sometimes see small skin imprints at the point of contact between the wire speculum and the eyelid, when we were interested whether this type of speculum would be less tolerated by patients in comparison to the speculum with a closed blades. On the other hand, a solid speculum could theoretically lead to greater glare due to greater light reflection from the closed blade. In group 3, where we changed the type of speculum, we did not notice any statistically significant effect on the perception of pain, pressure, lighting, or the duration of the operation.

In cases of perioperative fibrosis of the posterior capsule or in younger patients, we perform primary posterior CCC as a standard procedure at our clinic. We perform the procedure under an implanted IOL, without the use of a viscomaterial, using 23 G forceps. In subgroup 4, we wanted to find the influence of PPCCC on subjective perception of the surgery. In 1996, Galand et al. suggested PPCCC as a routine procedure during cataract surgery in adults [32]. Gibran et al. proposed a new technique for performing PPCCC under an implanted IOL [33]. Nowadays, PPCCC is widely used in paediatric cataract surgery in the hope of preventing PCO, but not routinely adopted in adult cataract surgery, attributing to concerns regarding associated complications, including vitreous interface damage, vitreous prolapse, resulting cystoid macular oedema, and even retinal detachment. Published studies have demonstrated the safety and efficacy of PPCCC in age-related cataract [34–36]. In our study, PPCCC had no effect on the patient's subjective perception. The duration of the surgery was objectively prolonged by an average of 18 seconds (294 ± 63 seconds in eyes with intact posterior capsule and 312 ± 68 seconds in eyes with PPCCC); however, this difference was not statistically significant and had no effect on the subjective perception of the duration of the surgery.

The last change whose effect on the subjective perception of cataract surgery we observed in subgroup 5 was communication with the patient. As patients are fully conscious during the procedure, it is important that they remain still. There are a variety of reasons why patients may need to move, and it is important that the surgeon is aware that this may happen. Some centres offer a nurse's hand or an electronic patient controller alert device [37]. It is also essential to choose the right words during the communication to avoid stressing patients. Interestingly, the data highlighted that some commonly used surgical terms such as “knife” and “scalpel” provoke considerable anxiety in the conscious patient. This varied according to age and sex, with younger and female patients being the most vulnerable. Other events identified as potential stressors, such as casual conversations and movements among theatre staff, were actually shown to be non-stressful and, in some cases, stress-relieving [38]. In our subgroup of patients, we failed to demonstrate that communication during the procedure had a greater effect on the subjective perception of the surgery.

A relatively interesting fact was the relatively high variability, especially in the perception of pain between subgroups of patients. The lowest pain was reported by patients in the subgroup where we examined the effect of anaesthetics (0.32 and 0.44, respectively). On the contrary, the greatest pain was reported by patients in the group where we examined the effect of communication (1.18 and 1.16, respectively). Given that the course of the operation was always identical except for the changed parameters, we explain this variability by composition of the group of patients, or other reasons that are unclear to us. However, since we compared the difference between one eye and the other eye of the same patient, this difference between the groups has no effect on the outcome.

## 5. Conclusion

First and second eye surgery within the SBCS is perceived similarly by patients (pain, pressure, glare, and perception of the length of the operation). It is therefore possible to use this fact to determine the effect of minor changes in the surgical protocol on the subjective perception of the operation. Thus, the SBCS can be used to optimize the parameters of cataract surgery. In our group of patients, we found a positive effect of intracameral anesthesia (Mydrane) on the perception of pain during surgery. We were unable to determine a statistically significant effect of light intensity, type of speculum, creation of PPCCC, and communication with the patient during surgery.

The main impact of this work is to draw attention to the possibility of using SCBS to optimize the parameters of cataract surgery. During simultaneous surgery of both eyes, where the operation on each eye was performed somewhat differently, the patient may very well describe any changes in the subjective perception of the operation. Unlike standard studies comparing different types of intervention inter-individually, this is an intra-individual comparison. As part of the research, the doctor can evaluate the effect of each step and possibly adjust his surgical procedure. Thus, for example, we have introduced standard intracameral anesthesia into routine practice in our departments.

## Figures and Tables

**Table 1:** Baseline and demographic data of the study population.

Parameter	All patients	Subgroup					
		Control	(1) Anesthesia	(2) Light	(3) Speculum	(4) PCCC	(5) Communication
Mean age (years)	72.5 ± 8.4	70.4 ± 7.5	73.2 ± 8.0	71.8 ± 8.9	72.3 ± 8.7	73.5 ± 7.4	73.7 ± 8.4
Male (*N* (%))	114 (38.0)	19 (38.0)	14 (28.0)	21 (42.0)	17 (34.0)	18 (36.0)	25 (50.0)
Female (*N* (%))	186 (62.0)	31 (62.0)	36 (72.0)	29 (58.0)	33 (66.0)	32 (64.0)	25 (50.0)
Eye (N)	600	100	100	100	100	100	100
OD (N)	300	50	50	50	50	50	50
OS (N)	300	50	50	50	50	50	50
Mean power of implanted IOL (D)	21.3 ± 3.2	20.5 ± 2.8	21.2 ± 3.3	21.3 ± 2.9	21.7 ± 3.4	22.2 ± 3.5	21.5 ± 2.7

**Table 2:** Perception of pain, pressure, and glare in the control subgroup (without change of any parameter between the right and left eye): mean ± SD, median (min-max); *p* values of Wilcoxon signed-rank test between right and left eye.

Eye	Pain		Pressure		Glare	
	Mean ± SD	Median (min–max)	Mean ± SD	Median (min–max)	Mean ± SD	Median (min–max)
Both eyes	0.72 ± 1.11	0 (0–5)	1.02 ± 0.85	1 (0–4)	1.12 ± 1.20	0 (0–4)
Right eye	0.70 ± 1.14	0 (0–5)	1.04 ± 0.92	1 (0–4)	1.10 ± 0.85	0 (0–4)
Left eye	0.74 ± 1.07	0 (0–5)	1.00 ± 0.77	1 (0–3)	1.14 ± 0.92	1 (0–4)
*p* value right vs. left	0.299		0.344		0.161	

**Table 3:** Comparison of perception of time of surgery in the control subgroup (without change of any parameter between right and left eye): mean ± SD; *p* values of paired *t*-test between right and left eye and between real and subjectively perceived time.

Eye	Real time (sec)	Subjectively perceived time (sec)	*p* value real vs. perceived
	Mean ± SD	Mean ± SD	
Both eyes	261.62 ± 74.79	255.90 ± 131.20	0.686
Right eye	260.76 ± 65.48	258.26 ± 124.98	0.903
Left eye	262.48 ± 83.81	253.54 ± 138.49	0.484
*p* value right vs. left	0.913	0.864	

**Table 4:** Comparison of perception of pain, pressure, and glare between eyes with and without changing parameters of surgery in 5 subgroups: mean ± SD, median (min-max); *p* values of Wilcoxon signed-rank test.

Subgroup	Pain		Pressure		Glare	
	Mean ± SD	Median (min–max)	Mean ± SD	Median (min–max)	Mean ± SD	Median (min–max)
*(1) Anesthesia*						
Only topic	0.44 ± 0.81	0 (0–3)	0.60 ± 0.70	0 (0–3)	0.62 ± 1.01	0 (0–3)
Intracameral	0.32 ± 0.71	0 (0–3)	0.52 ± 0.58	0 (0–2)	0.62 ± 1.05	0 (0–4)
*p* value topic vs. intr.	**0.032** ^*∗*^		0.351		1.000	

*(2) Light intensity*						
40%	0.80 ± 1.50	0 (0–7)	0.88 ± 0.96	0 (0–3)	0.82 ± 1.10	0 (0–4)
80%	0.92 ± 1.83	0 (0–10)	0.82 ± 1.02	0.5 (0–4)	0.86 ± 1.11	0.5 (0–4)
*p* value 40% vs. 80%	0.444		0.537		0.598	

*(3) Speculums*						
Solid	0.49 ± 0.83	0 (0–3)	0.57 ± 0.67	0 (0–2)	0.67 ± 1.09	0 (0–4)
Wire	0.50 ± 0.73	0 (0–2)	0.47 ± 0.58	0 (0–2)	0.61 ± 1.06	0 (0–4)
*p* value solid vs. wire	0.837		0.301		0.083	

*(4) PPCCC*						
Intact	0.50 ± 1.28	0 (0–6)	0.90 ± 0.93	1 (0–3)	0.78 ± 1.13	0 (0–3)
PPCCC	0.46 ± 1.05	0 (0–8)	0.94 ± 0.91	1 (0–3)	0.72 ± 0.99	0 (0–4)
*p* value intact vs. PPCCC	0.659		0.699		0.444	

*(5) Communication*						
No	1.18 ± 1.85	0 (0–7)	1.00 ± 1.18	1 (0–4)	1.30 ± 1.25	1 (0–4)
Speaking	1.16 ± 1.844	0 (0–10)	1.00 ± 1.16	1 (0–4)	1.30 ± 1.27	1 (0–4)
*p* value no vs. speaking	0.932		1.000		1.000	

^*∗*^Statistically significant results are marked in bold.

**Table 5:** Comparison of perception of time between eyes with and without changing parameters of surgery in 5 subgroups: mean ± SD; *p* values of paired *t*-test between real and subjectively perceived time.

Subgroup	Real time (sec)	Subjectively perceived time (sec)	*p* value real vs. perceived
	Mean ± SD	Mean ± SD	
*(1) Anesthesia*			
Only topic	268.82 ± 60.23	234.30 ± 90.96	**0.027** *∗*
Intracameral	266.56 ± 44.47	234.68 ± 89.17	**0.028** *∗*
*p* value topic vs. intracameral	0.831	0.983	

*(2) Light intensity*			
40%	281.96 ± 58.96	296.04 ± 143.45	0.457
80%	297.58 ± 70.95	286.50 ± 160.48	0.530
*p* value 40% vs. 80%	0.234	0.755	

*(3) Speculums*			
Solid	275.52 ± 49.76	276.36 ± 104.62	0.589
Wire	283.36 ± 56.86	272.20 ± 98.71	0.470
*p* value solid vs. wire	0.465	0.812	

*(4) PPCCC*			
Intact	290.68 ± 64.98	310.26 ± 147.52	0.368
PPCCC	312.36 ± 70.04	314.40 ± 155.85	0.931
*p* value intact vs. PPCCC	0.112	0.892	

*(5) Communication*			
No	303.10 ± 85.15	339.22 ± 138.56	0.111
Speaking	292.44 ± 97.45	232.32 ± 180.52	0.193
*p* value no vs. speaking	0.562	0.622	

^*∗*^Statistically significant results are marked in bold.

## Data Availability

The data are available on request to the first author (studenypavel@seznam.cz).
